# Estimation of evolutionary parameters using short, random and partial sequences from mixed samples of anonymous individuals

**DOI:** 10.1186/s12859-015-0810-y

**Published:** 2015-11-04

**Authors:** Steven H. Wu, Allen G. Rodrigo

**Affiliations:** 1 Biodesign Institute, Arizona State University, Tempe, AZ 85287 USA; 2 Department of Biology, Duke University, Box 90338, Durham, NC 27708 USA; 3 The National Evolutionary Synthesis Center, Durham, NC 27705 USA

**Keywords:** Markov chain Monte Carlo, Next generation sequencing, Short read sequences, Approximate Bayesian computation, Evolutionary genetics

## Abstract

**Background:**

Over the last decade, next generation sequencing (NGS) has become widely available, and is now the sequencing technology of choice for most researchers. Nonetheless, NGS presents a challenge for the evolutionary biologists who wish to estimate evolutionary genetic parameters from a mixed sample of unlabelled or untagged individuals, especially when the reconstruction of full length haplotypes can be unreliable. We propose two novel approaches, least squares estimation (LS) and Approximate Bayesian Computation Markov chain Monte Carlo estimation (ABC-MCMC), to infer evolutionary genetic parameters from a collection of short-read sequences obtained from a mixed sample of anonymous DNA using the frequencies of nucleotides at each site only without reconstructing the full-length alignment nor the phylogeny.

**Results:**

We used simulations to evaluate the performance of these algorithms, and our results demonstrate that LS performs poorly because bootstrap 95 % Confidence Intervals (CIs) tend to under- or over-estimate the true values of the parameters. In contrast, ABC-MCMC 95 % Highest Posterior Density (HPD) intervals recovered from ABC-MCMC enclosed the true parameter values with a rate approximately equivalent to that obtained using BEAST, a program that implements a Bayesian MCMC estimation of evolutionary parameters using full-length sequences. Because there is a loss of information with the use of sitewise nucleotide frequencies alone, the ABC-MCMC 95 % HPDs are larger than those obtained by BEAST.

**Conclusion:**

We propose two novel algorithms to estimate evolutionary genetic parameters based on the proportion of each nucleotide. The LS method cannot be recommended as a standalone method for evolutionary parameter estimation. On the other hand, parameters recovered by ABC-MCMC are comparable to those obtained using BEAST, but with larger 95 % HPDs. One major advantage of ABC-MCMC is that computational time scales linearly with the number of short-read sequences, and is independent of the number of full-length sequences in the original data. This allows us to perform the analysis on NGS datasets with large numbers of short read fragments. The source code for ABC-MCMC is available at https://github.com/stevenhwu/SF-ABC.

## Background

Over the last decade, next generation sequencing (NGS) has become widely available, and is now the sequencing technology of choice for most researchers. NGS produces sequences that are relatively short, varying between 50 bp to 400 bp depending on the specific platform [1–3]. Researchers use NGS in several different ways. In this manuscript, we consider the use of NGS in evolutionary studies, where short read fragments are obtained from longer, amplified target sequences in mixed samples of unlabeled or untagged (= “anonymous”) individuals. These types of samples are often collected from viral or bacterial populations. The traditional sampling protocol for evolutionary studies that rely on sequences from many individuals has been to use Sanger sequencing technology to obtain the sequence(s) of one (or more) DNA fragment(s) from each individual in the sample separately. This is typically followed by a multiple sequence alignment and reconstruction of the phylogeny or genealogy, perhaps with the simultaneous inference of relevant evolutionary parameters [4, 5].

With NGS, short read fragments are typically shorter than the fragment of interest. When NGS is applied to a mixed collection of DNA from several individuals, the challenge for the evolutionary biologist is the absence of an alignment of full-length sequences, each corresponding to an individual in the sample [6]. And without an alignment of full-length sequences, how does one estimate the evolutionary parameters of interest?

One approach is to attempt to reconstruct the full length haplotypes, and use an alignment of these reconstructed haplotypes in standard evolutionary analyses [7–9]. There are several programs that attempt to reconstruct the full-length haplotypes from short read fragments obtained from mixed, unlabeled, collections of individuals e.g., ShoRAH, ViSpA, PredictHaplo and Qure [10–13]. Analyses have shown that with many data sets, reconstruction of haplotypes can be unreliable, producing either too many haplotypes and/or sequences that have relatively low identity to the original sequences [14–16]. Consequently, a researcher who chooses to use these reconstructed full-length haplotypes with any program that requires full-length alignments, will be implicitly integrating the errors of haplotype reconstruction into their estimation of evolutionary parameters.

We propose two alternative approaches to infer population genetic parameters from a collection of short-read sequences obtained from a mixed sample of anonymous DNA using the frequencies of nucleotides at each site only. To our knowledge, there is no existing method capable of estimating these parameters without reconstructing the full length sequence alignment nor the phylogeny. A similar approach had been proposed by Johnson and Slatkin previously [17], but their method focuses on samples of very large numbers of individuals where each read in an alignment of short-reads is assumed to come from a separate genome. In contrast, the methods we propose assumes either (1) that a relatively short fragment of the genome is the target of NGS, and/or (2) there are relatively few individual organisms in the sample. In essence, these assumptions ensure that each site for each individual is covered by multiple reads, so that the frequency of nucleotides at each site can be estimated with reasonable accuracy. In samples of viruses and bacteria, for instance, the amplified region is small, say, a few kilobases long, and the number of genomes in a single PCR reaction is often unknown ranging from the tens to the thousands. With microbial populations, typically viruses, there is also the opportunity to collect serial samples from the same fast evolving species over a period of time. For this reason, the methods we have developed estimate the population genetic parameters *θ* ∝ *Nμ*, the effective population size scaled by mutation rate, and *μ*, the mutation rate per site per unit time [18, 19].

We describe two algorithms to estimate these parameters, Least Squares (LS) estimation [20] and Approximate Bayesian Computation Markov chain Monte Carlo (ABC-MCMC) estimation [21–23]. One advantage of the ABC-MCMC approach over more traditional MCMC approaches is that ABC-MCMC does not require formulation or computation of a likelihood; instead, the method relies on the use of summary statistics derived from simulated data to accept or reject a proposal in the Markov chain. The details of these two algorithms are described in the next section.

We used simulations to evaluate the performance of these algorithms, and we compared these to the results obtained with BEAST [8], a program that is commonly used to simultaneously infer phylogenies and evolutionary parameters using Bayesian MCMC inference with full-length sequences. Our simulations demonstrate that LS point estimates are unbiased, but produce bootstrap intervals that typically over- or underestimate true parameter values. In contrast, ABC-MCMC is able to estimate evolutionary parameters without reconstructing full-length haplotypes, producing 95 % Highest Posterior Density (95 % HPD) intervals that have equivalent coverage to those obtained by MCMC with full-length alignments; however, there can be up to a 10-fold difference between the lowest and highest bound of each 95 % HPD.

## Methods

As noted above, both algorithms apply to samples of short-read sequences obtained from a collection of longer target sequences from mixed and unlabeled individuals in a population. The first method is based on least squares (LS) estimation. As we show below, the second method applies the LS results as a pre-processing step prior to beginning the ABC-MCMC. Both methods estimate evolutionary parameters using only the proportion of each nucleotide at each site, and do not require reconstruction of full length haplotypes nor the phylogeny/genealogy. If serial samples are available, sequences from a later timepoint share common ancestors with those from the earlier timepoint. We assume that samples collected from each timepoint are sequenced separately with NGS technology.

As we noted earlier, we assume that each short read sequenced by NGS will be shorter than the full length haplotypes, and we will obtain many more short read fragments than the original number of haplotypes. We also assume that a reference or consensus sequence for the targeted region is available, and we are able to align each short read fragment to a unique location on the reference sequence. After the short reads are aligned to the reference, we can count the frequency of each nucleotide at each site. In practice, the frequency of each nucleotide will be influenced by the sequencing error from NGS [1]. For the simplicity of the algorithms, we assume that the short-reads have been error-corrected prior to analysis.

### Least squares (LS) estimation

For serial samples, we can estimate the intra-timepoint (within a single sample) and inter-timepoint (between samples from two different timepoints) average pairwise sequence diversity. Both inter-timepoint diversity (*D*_*inter*_) and intra-timepoint diversity (*D*_*intra*_) are calculated using the proportion of each nucleotide at each site. The intra-timepoint diversity (*D*_*intra,s,t*_) for site *s* at time *t* is calculated as:

1 $$ {D}_{intra,s,t}=1-{\displaystyle \sum_{j\in A,C,G,T}}{F_{s,j,t}}^2 $$

where *F*_*s,j,t*_ is the proportion of nucleotide *j* at site *s* at time *t.*

Similarly, the inter-timepoint diversity (*D*_*inter,s,t1,t2*_) between time *t*_*1*_ and *t*_*2*_ at site *s* is calculated as

2 $$ {D}_{inter,s,t1,t2}=1-{\displaystyle \sum_{j\in A,C,G,T}}{F}_{s,j,t1}{F}_{s,j,t2} $$

Once the average pairwise diversity for each site is calculated, the mean intra-timepoint diversity for any specified timepoint is given by:

3 $$ {D}_{intra,t}=\frac{1}{n}{\displaystyle \sum_{s=1}^n}{D}_{intra,s,t} $$

and the mean inter-timepoint diversity between time *t*_*1*_ and *t*_*2*_ is:

4 $$ {D}_{inter,t1,t2}=\frac{1}{n}{\displaystyle \sum_{s=1}^n}{D}_{inter,s,t1,t2} $$

where *n* is the number of sites. If the sequences are obtained from *T* timepoints, there will be *T* × (*T* − 1)/2 estimates of average diversity, of which *T* will be intra-timepoint diversities.

The LS method uses both inter- and intra-timepoint diversity to estimate effective population size and mutation rate, based on the method described by [20]. Population genetics tells us that in any given sample from a constant-sized population of a set of sequences of a neutrally evolving locus, average pairwise sequence diversity (measured as the average proportional distance between any two sequences in the sample) is an estimate of *θ*, which is proportional to the product of mutation rate and effective population size, with the proportionality constant determined by whether the population is haploid (proportionality constant = 2, *θ* = 2*Nμ*) or diploid (proportionality constant = 4, *θ* = 4*Nμ*).

To estimate the parameters of interest, let *μ* be the mutation rate per unit of time, *θ* be the effective population size scaled by *μ*, and *Δt* the time between two sampling events. Note that both *μ* and *Δt* are scaled to the same unit of time; typically this will be chronological time but, rarely, time in generations may be available. Under a constant population size, and a constant mutation rate, there are only two parameters to be estimated, *θ* and *μ*. As noted above, *θ* is estimated by *D*_*intra*_ and *θ* + *μ∆t* is estimated by *D*_*inter*_. Once estimates of *θ* and *μ* are obtained, we can estimate *kN* = *θ*/*μ*, where *k* is the unspecified proportionality constant.

We can construct a least squares regression by letting *Y* be a vector of all *D*_*inter*_ and *D*_*intra*_, and *X* be an indicator variable that identifies whether/how *θ* and *μ* contribute to the expectation of *D*_*inter*_ or *D*_*intra*_. For the constant population, constant mutation rate model, the indicator value for *θ* is always 1 and the indicator of *μ* is just *∆t.* We are then able to fit **Y** *=* **XΒ** using least-squares, where the **Β** is the LS estimator of parameter *θ* and *μ*. For example, if there are three timepoints *T*_*A*_, *T*_*B*_, and *T*_*C*_, and the intervals between each adjacent pair is 200 units of time apart, we can construct a set of linear equations to express the relationship between these parameters, as follows:

5 $$ \begin{array}{ccc}\hfill \begin{array}{c}\hfill {D}_{intra-A\ }\ \hfill \\ {}\hfill {D}_{intra-B\kern0.5em }\hfill \\ {}\hfill {D}_{intra-C\kern0.5em }\ \hfill \end{array}\hfill & \hfill \begin{array}{c}\hfill =\hfill \\ {}\hfill =\hfill \\ {}\hfill =\hfill \end{array}\hfill & \hfill \begin{array}{c}\hfill \theta \hfill \\ {}\hfill \theta \hfill \\ {}\hfill \theta \hfill \end{array}\hfill \\ {}\hfill \begin{array}{c}\hfill {D}_{inter- AB}\hfill \\ {}\hfill {D}_{inter-BC}\hfill \\ {}\hfill {D}_{inter- AC}\hfill \end{array}\hfill & \hfill \begin{array}{c}\hfill =\hfill \\ {}\hfill =\hfill \\ {}\hfill =\hfill \end{array}\hfill & \hfill \begin{array}{c}\hfill \theta +200\mu \hfill \\ {}\hfill \theta +200\mu \hfill \\ {}\hfill \theta +400\mu \hfill \end{array}\hfill \end{array} $$

The linear equations can be shown as **Y** = **XB**, where

6 $$ \begin{array}{l}\mathbf{Y}' = \left[{D}_{intra-A},\ {D}_{Intra-B},\ {D}_{intra-C},{D}_{inter- AB},{D}_{inter-BC},{D}_{inter- AC}\right]\\ {}\boldsymbol{X}=\left[\begin{array}{cc}\hfill \begin{array}{c}\hfill 1\hfill \\ {}\hfill 1\hfill \\ {}\hfill 1\hfill \end{array}\hfill & \hfill \begin{array}{c}\hfill 0\hfill \\ {}\hfill 0\hfill \\ {}\hfill 0\hfill \end{array}\hfill \\ {}\hfill \begin{array}{c}\hfill 1\hfill \\ {}\hfill 1\hfill \\ {}\hfill 1\hfill \end{array}\hfill & \hfill \begin{array}{c}\hfill 200\hfill \\ {}\hfill 200\hfill \\ {}\hfill 400\hfill \end{array}\hfill \end{array}\right]\\ {}\kern16em \mathbf{B}'=\kern1em \left[\theta \kern1.5em \mu \right]\end{array} $$

Using the least squares method to solve for **B = (X’X)** ^**−1**^ **X’Y**, we obtain our estimation of *θ* and *μ*.

The model can be extended to multiple population sizes and/or multiple mutation rates. If there are three timepoints *T*_*A*_, *T*_*B*_, and *T*_*C*_, then we can estimate the effective population size for each timepoint using the intra distance within each timepoint. Let *θ*_*A*_, *θ*_*B*_, and *θ*_C_ be the scaled population sizes for timepoint *T*_*A*_, *T*_*B*_ and *T*_*C*_. *Δ*_*AB*_ is the time difference between timepoint *T*_*A*_ and *T*_B_, and *Δ*_*BC*_ is the time difference between timepoint *T*_*B*_ and *T*_C_.

7 $$ \begin{array}{ccc}\hfill \begin{array}{c}\hfill {D}_{intra-A\ }\ \hfill \\ {}\hfill {D}_{intra-B\kern0.5em }\hfill \\ {}\hfill {D}_{intra-C\kern0.5em }\ \hfill \end{array}\hfill & \hfill \begin{array}{c}\hfill =\hfill \\ {}\hfill =\hfill \\ {}\hfill =\hfill \end{array}\hfill & \hfill \begin{array}{c}\hfill {\uptheta}_{\mathrm{A}}\hfill \\ {}\hfill {\uptheta}_{\mathrm{B}}\hfill \\ {}\hfill {\uptheta}_{\mathrm{C}}\hfill \end{array}\hfill \\ {}\hfill \begin{array}{c}\hfill {D}_{inter- AB}\hfill \\ {}\hfill {D}_{inter-BC}\hfill \\ {}\hfill {D}_{inter- AC}\hfill \end{array}\hfill & \hfill \begin{array}{c}\hfill =\hfill \\ {}\hfill =\hfill \\ {}\hfill =\hfill \end{array}\hfill & \hfill \begin{array}{c}\hfill {\theta}_A+\mu {\varDelta}_{AB}\hfill \\ {}\hfill {\theta}_B+\mu {\varDelta}_{\boldsymbol{B}C}\hfill \\ {}\hfill {\theta}_A+\mu {\varDelta}_{AB}+\mu {\varDelta}_{BC}\hfill \end{array}\hfill \end{array} $$

Therefore we update the matrix

8 $$ \begin{array}{l}\mathbf{X}=\left[\begin{array}{cc}\hfill \begin{array}{ccc}\hfill 1\hfill & \hfill 0\hfill & \hfill 0\hfill \\ {}\hfill 0\hfill & \hfill 1\hfill & \hfill 0\hfill \\ {}\hfill 0\hfill & \hfill 0\hfill & \hfill 1\hfill \end{array}\hfill & \hfill \begin{array}{c}\hfill 0\hfill \\ {}\hfill 0\hfill \\ {}\hfill 0\hfill \end{array}\hfill \\ {}\hfill \begin{array}{ccc}\hfill 1\hfill & \hfill 0\hfill & \hfill 0\hfill \\ {}\hfill 0\hfill & \hfill 1\hfill & \hfill 0\hfill \\ {}\hfill 1\hfill & \hfill 0\hfill & \hfill 0\hfill \end{array}\hfill & \hfill \begin{array}{c}\hfill {\varDelta}_{AB}\hfill \\ {}\hfill {\varDelta}_{BC}\hfill \\ {}\hfill {\varDelta}_{AB}+{\varDelta}_{BC}\hfill \end{array}\hfill \end{array}\right]\\ {}\kern1.5em \mathbf{B}\hbox{'}=\left[\begin{array}{cc}\hfill \begin{array}{ccc}\hfill {\theta}_A\hfill & \hfill {\theta}_B\hfill & \hfill {\theta}_C\hfill \end{array}\hfill & \hfill \mu \hfill \end{array}\right]\end{array} $$

Alternatively, for a constant population size and multiple mutation rates, the model can be specific as:

9 $$ \begin{array}{ccc}\hfill \begin{array}{c}\hfill {D}_{intra-A\ }\ \hfill \\ {}\hfill {D}_{intra-B\kern0.5em }\hfill \\ {}\hfill {D}_{intra-C\kern0.5em }\ \hfill \end{array}\hfill & \hfill \begin{array}{c}\hfill =\hfill \\ {}\hfill =\hfill \\ {}\hfill =\hfill \end{array}\hfill & \hfill \begin{array}{c}\hfill \theta \hfill \\ {}\hfill \theta \hfill \\ {}\hfill \theta \hfill \end{array}\hfill \\ {}\hfill \begin{array}{c}\hfill {D}_{inter- AB}\hfill \\ {}\hfill {D}_{inter-BC}\hfill \\ {}\hfill {D}_{inter- AC}\hfill \end{array}\hfill & \hfill \begin{array}{c}\hfill =\hfill \\ {}\hfill =\hfill \\ {}\hfill =\hfill \end{array}\hfill & \hfill \begin{array}{c}\hfill \theta +{\mu}_{AB}{\varDelta}_{AB}\hfill \\ {}\hfill \theta +{\mu}_{BC}{\varDelta}_{\boldsymbol{B}C}\hfill \\ {}\hfill \theta +{\mu}_{AB}{\varDelta}_{AB}+{\mu}_{BC}{\varDelta}_{BC}\hfill \end{array}\hfill \end{array} $$

Therefore we update the matrices **X** and **B** as

10 $$ \begin{array}{l}\mathbf{X}=\left[\begin{array}{cc}\hfill \begin{array}{c}\hfill 1\hfill \\ {}\hfill 1\hfill \\ {}\hfill 1\hfill \end{array}\hfill & \hfill \begin{array}{cc}\hfill 0\hfill & \hfill 0\hfill \\ {}\hfill 0\hfill & \hfill 0\hfill \\ {}\hfill 0\hfill & \hfill 0\hfill \end{array}\hfill \\ {}\hfill \begin{array}{c}\hfill 1\hfill \\ {}\hfill 1\hfill \\ {}\hfill 1\hfill \end{array}\hfill & \hfill \begin{array}{cc}\hfill {\varDelta}_{AB}\hfill & \hfill 0\hfill \\ {}\hfill 0\hfill & \hfill {\varDelta}_{BC}\hfill \\ {}\hfill {\varDelta}_{AB}\hfill & \hfill {\varDelta}_{BC}\hfill \end{array}\hfill \end{array}\right]\\ {}\mathbf{B}\hbox{'}=\left[\begin{array}{cc}\hfill \theta \hfill & \hfill \begin{array}{cc}\hfill {\mu}_{AB}\hfill & \hfill {\mu}_{BC}\hfill \end{array}\hfill \end{array}\right]\end{array} $$

To obtain confidence intervals for our estimates, we used a bootstrap procedure in which we generated 1000 pseudoreplicate datasets by resampling sites with replacement along the alignment of short-read sequences. We did this separately for each timepoint in our simulated datasets. For each pseudoreplicate we recalculated the site frequencies, *F*_*s,j,t,*_, and we were able to estimate *N* and *μ*; 95 % Confidence Intervals were obtained by taking values corresponding to upper and lower 2.5 % percentiles of ordered bootstrap estimates.

### ABC-MCMC (Approximate Bayesian Computation - Markov chain Monte Carlo)

Markov chain Monte Carlo Bayesian inference is a computationally intensive technique for recovering the posterior probability of parameters of interest, while taking account of prior knowledge (including the degree of uncertainty) about these parameters. If *P*(*D*|**ϕ**) (often referred to as the likelihood of **ϕ**), is the probability of obtaining the data given a set of parameters, **ϕ**, and *P*(**ϕ**) is the prior information we have about the joint distribution of these parameters, then the posterior distribution, *P*(**ϕ**|*D*), is proportional to the product of likelihood and prior, *P*(**ϕ**|*D*) ∝ *P*(*D*|**ϕ**)*P*(**ϕ**). Often it is very difficult to obtain the posterior distribution function analytically. The elegance of MCMC resides in its ability to derive the relative posterior distribution by using a proposal distribution to randomly generate a Markov chain of potential states (= values that the parameters of interest can take), and sampling from this chain by accepting or rejecting states in proportion to the posterior density. MCMC works because although it is not easy to calculate the distribution of *P*(**ϕ**|*D*), it is easy to obtain *P*(ϕ*|*D*), for a specific value of ϕ*. Consequently by repeatedly sampling ϕ* in the correct proportions, the distribution of *P*(**ϕ**|*D*) is approximated. Once a sample of sufficient size is obtained, it becomes possible to derive estimates for the parameters of interest.

One common implementation of MCMC uses the Metropolis-Hastings algorithm [24, 25], which can be described by the following steps.

Step 1: Begin with initial parameter values **ϕ** ^***i***^.
Step 2: Propose a new parameter value ϕ* using the proposal distribution *q*(ϕ*|**ϕ** ^***i***^).
Step 3: Calculate the acceptance ratio, *α*, using the following formula:
  $$ \alpha = min\left\{1,\frac{P\left({\boldsymbol{\upphi}}^{*}\Big|D\right)q\left({\boldsymbol{\upphi}}^{\boldsymbol{i}}\Big|{\boldsymbol{\upphi}}^{*}\right)}{P\left({\boldsymbol{\upphi}}^{\boldsymbol{i}}\Big|D\right)q\left({\boldsymbol{\upphi}}^{*}\Big|{\boldsymbol{\upphi}}^{\boldsymbol{i}}\right)}\right\} $$
  Generate μ from *U*(0, 1) and accept **ϕ** ^***i*** + 1^ = ϕ* if *µ* < *α*.
  Otherwise set **ϕ** ^***i*** + 1^ = **ϕ** ^***i***^.
Step 4 Set *i* = *i* + 1 and repeat Step 1.

The algorithm is repeated until the Markov chain samples from the target distribution, typically the (joint) posterior distribution of the parameter(s).

Approximate Bayesian Computation (ABC) is a simulation-based algorithm for Bayesian inference [21–23]. ABC does not require the calculation of the likelihood *P*(*D*|**ϕ**); instead, it uses the agreement between summary statistics obtained from *D*, and those obtained from simulations of data under different values of **ϕ** to obtain the relative posterior probability distribution *P*(**ϕ**|*D*). If the summary statistic, *S*, is (nearly) sufficient then *P*(*D*|**ϕ**, *S*) ≅ *P*(*D*|*S*). ABC is used precisely because it circumvents the need to calculate a challenging or intractable likelihood. Here, we describe our ABC-MCMC procedure as follows:

1. Calculate a set of *p* sufficient statistics, ***S*** ^*o*^ = (*S*_1_^*o*^, ⋯, *S*_*p*_^*o*^), on the observed dataset, *D* ^*o*^.
2. If *i* = 1, draw the initial parameter value **ϕ** ^***i***^ from prior distribution *P*(**ϕ**). If *i* > 1, then propose the new parameter value ϕ* from *q*(ϕ* | **ϕ** ^***i***^), where *q*(. | **ϕ** ^***i***^) is the proposal distribution with mean equal to **ϕ** ^***i***^.
3. Simulate the dataset (D*) using the model parameter values ϕ* and calculate the sufficient statistics S* = (*S*_1_^*^, ⋯, *S*_p_^*^) based on D*. If the distance between the sufficient statistics on the simulated dataset and the observed statistics, *d*(S*, ***S*** ^*o*^)*,* is greater than a threshold *ε*, then reject the proposed parameter values and set **ϕ** ^***i*** + 1^ = **ϕ** ^***i***^. If the difference is less than *ε*, then set **ϕ** ^***i*** + 1^ = ϕ* with probability *α*, calculated by the modified Metropolis-Hasting ratio:
  $$ \alpha = min\left\{1,\ \frac{q\left({\boldsymbol{\upphi}}^{\boldsymbol{i}}\Big|{\boldsymbol{\upphi}}^{*}\right)P\left({\boldsymbol{\upphi}}^{*}\right)}{q\left({\boldsymbol{\upphi}}^{*}\Big|{\boldsymbol{\upphi}}^{\boldsymbol{i}}\right)P\left({\boldsymbol{\upphi}}^{\boldsymbol{i}}\right)}\right\} $$
  The distance, *d*(S*, ***S*** ^*o*^), is calculated as:
  $$ d\left({\boldsymbol{S}}^{*},{\boldsymbol{S}}^o\right)={\displaystyle \sum_{j=1}^p}\frac{{\left({S^{*}}_j-{S^o}_j\right)}^2}{{S^o}_j} $$
4. Set *i* = *i* + 1 and go back to step 2 for a large number of iterations.

Although ABC requires that sufficient statistics (or nearly sufficient statistics) are used, it is non-trivial obtaining appropriate sufficient statistics. For this reason, Fearnhead et al. [26] proposed a semi-automatic method to generate “nearly” sufficient statistics for ABC by using a linear combination of commonly-used summary statistics. The linear combination is obtained by regressing the summary statistics against known values of **ϕ** from simulated training datasets. The regression equation serves as the new summary statistic. The outline of their algorithm is as follows:

1. Define a training region in parameter space that is representative of the parameter values one expects to obtain in relatively high densities in the posterior distribution.
2. Draw parameter value **ϕ** ^***T***^ from this training region and simulate a dataset, *D* ^*T*^, based on **ϕ** ^***T***^. Calculate *p* summary statistics, ***S*** ^*T*^ = (*S*_1_^*T*^, ⋯, *S*_*p*_^*T*^), on *D* ^*T*^. There are no hard-and-fast rules about which summary statistics to use, but for our data, there are obvious candidates, including average pairwise intra- and inter-sample diversity, number of variable sites etc. (see below for a list of summary statistics used).
3. Repeat Step 2 for *k* iterations, where *k* ≫ *n*. Therefore, there are *k* values of **ϕ** ^***T***^ drawn from the training region, and for each **ϕ** ^***T***^ there are a set of *n* summary statistics.
4. For each parameter, *ϕ* ^*T*^ ∈ **ϕ** ^***T***^, regress the values of *ϕ* ^*T*^ against the set of summary statistics, ***S*** ^*T*^, obtained from all simulations. As noted, the LS regression equations that are obtained are a linear combination of summary statistics in **S**, and serves as a single-valued sufficient statistic for each *ϕ* ∈ **ϕ**, and used as S* in the ABC-MCMC algorithm above.

### Implementation of the full algorithm

For each dataset, we calculate the proportion of each nucleotide at each site of the alignment of short-read sequences to a reference sequence. Our LS estimation procedure is applied to obtain point estimates of the parameters of interest. These point estimates are used as guidelines to help us define the training region for Fearnhead et al.’s algorithm. We set the training region as a uniform distribution with mean equal to the LS estimate with upper and lower bounds set to 5 fold above and below the mean, i.e. from (0.2× to 5×).

Linear combinations of the following summary statistics are used as sufficient statistics in the regression. All summary statistics are calculated using the proportion of each nucleotide at each site.

1. Mean sequence distances, measured within each timepoint and between each pair of timepoints using Equations 3 and 4.
2. Variances for the sequence distances between each site within each timepoint and between each pair of timepoints.
  11 $$ Va{r}_{intra,t}=\frac{{\displaystyle {\sum}_{s=1}^n}{\left({D}_{intra,s,t}-\overline{D_{intra,t}}\right)}^2}{n} $$
  12 $$ Va{r}_{inter,t1,t2}=\frac{{\displaystyle {\sum}_{s=1}^n}{\left({D}_{inter,s,t1,t2}-\overline{D_{inter,t1,t2}}\right)}^2}{n} $$
3. A chi-squared distance is calculated with the following steps:
  - Setup 20 frequency categories at 0.05 intervals ranging from 0 to 1.
  - For each time point, assign each site to a frequency category that encompasses the proportion of the most frequent nucleotide for that site, and repeat this for all sites.
  - Finally, calculate the proportion of sites in each category to obtain a relative frequency spectrum ${FS}_t$. We also create a reference frequency spectrum $F{S}_R$, in which all categories have the same proportion, i.e. 0.05 each. Calculate the chi-square distance between the reference frequency spectrum and the frequency spectrum for each time point using the following formula,
  13 $$ {\displaystyle \sum_{category\ (c)=1}^{20}}\frac{{\left(F{S}_{t,c} - F{S}_{R,c}\right)}^2}{F{S}_{R,c}} $$
4. Divide the pattern of the proportion of each nucleotide between any two timepoints into four different categories, and record the number of sites in each category:
  - Category 1: Sites are identical across both timepoints.
  - Category 2: Sites in which one nucleotide is fixed in one timepoint, and the same nucleotide is in the majority in the other timepoint.
  - Category 3: There are mutations in both timepoints but the same nucleotide is most frequent in both timepoints (in other words, the nucleotide with the highest frequency in one timepoint is also the nucleotide with the highest frequency in the second timepoint).
  - Category 4: All others sites.

By applying the Fearnhead et al. algorithm, we obtain linear equations as sufficient statistics for *N* and *μ,* these are used in the ABC-MCMC above.

**Fig. 1:**
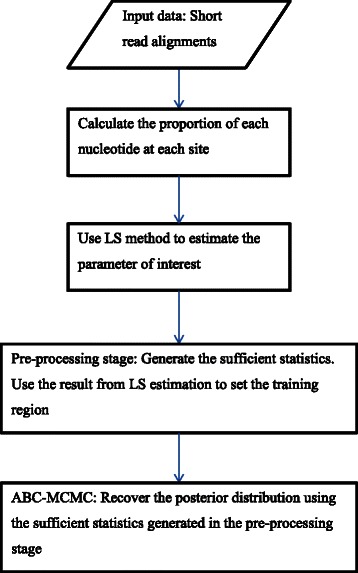
Flow chart of the full ABC-MCMC algorithm

Two priors are specified for ABC-MCMC: the prior distribution for *N*, the effective population size, is $p(N) = \frac{1}{N}$, and, the prior for mutation rate, *μ*, is a uniform distribution between [0,1]*.* The full algorithm is summarized in Fig. 1.

We have found that allowing *N* and *μ* to vary independently along the MCMC chain results in inefficient mixing, with higher autorcorrelation between samples of a given interval. For this reason, we used block updating, where both *N* and *μ* are updated at each generation of the chain [27, 28].

### Simulation analysis

Two sets of simulation analyses were performed. The first set tested the performance of the LS method with a range of evolutionary parameters. One hundred datasets were simulated by Bayesian Serial SimCoal [29]. The effective population size was fixed at 3000 and mutation rate fixed at 10^−5^ mutations per site per generation. By exploring different combinations of other parameters, we were able to estimate the performance of this algorithm. The number of sequences per timepoint were set to 5, 10, 20 and 40. We used 3, 5 and 10 timepoints in our simulations, with 100, 200, 400 and 600 generations between each time point. Tables 1, 2 and 3 show the combination of parameters used in the simulations. For each combination, 100 datasets were simulated.

**Table 1:** LS results with different number of sequences

No. of timepoints	No. of sequences per timepoints	No. of generations	Mean mutation rate (95 % confidence interval)	Mean effective population size (95 % confidence interval)
5	5	200	2.45E-05 (2.16E-05, 2.75E-05)	1245 (1084, 1406)
5	10	200	2.09E-05 (1.76E-05, 2.41E-05)	1852 (1537, 2168)
5	20	200	1.41E-05 (1.19E-05, 1.62E-05)	3483 (2146, 4820)
5	40	200	1.13E-05 (9.83E-06, 1.27E-05)	3311 (2781, 3841)

**Table 2:** LS results with different number of generations between timepoints

No. of timepoints	No. of sequences per timepoint	No. of generations	Mean mutation rate (95 % confidence interval)	Mean effective population size (95 % confidence interval)
5	40	100	1.59E-05 (1.37E-05, 1.8E-05)	2388 (1919, 2858)
5	40	200	1.13E-05 9.83E-06, 1.27E-05)	3311 (2781, 3841)
5	40	400	1.11E-05 (9.6E-06, 1.26E-05)	2897 (2573, 3221)
5	40	600	9.57E-06 (8.4E-06, 1.07E-05)	3429 (3016, 3842)

**Table 3:** LS results with different number of timepoints

No. of timepoints	No. of sequences per timepoint	No. of generations	Mean mutation rate (95 % confidence interval)	Mean effective population size (95 % confidence interval)
3	40	100	2.47E-05 (1.97E-05, 2.96E-05)	2286 (1647, 2924)
3	40	200	1.43E-05 (1.21E-05, 1.65E-05)	3276 (2601, 3950)
3	40	400	1.11E-05 (9.52E-06, 1.28E-05)	3472 (2981, 3962)
5	40	100	1.59E-05 (1.37E-05, 1.8E-05)	2388 (1919, 2858)
5	40	200	1.13E-05 (9.83E-06, 1.27E-05)	3311 (2781, 3841)
5	40	400	1.11E-05 (9.6E-06, 1.26E-05)	2897 (2573, 3221)
10	40	100	1.67E-05 (1.38E-05, 1.96E-05)	2591 (2224, 2958)
10	40	200	1.12E-05 (9.88E-06, 1.25E-05)	2949 (2560, 3338)
10	40	400	9.04E-06 (8.32E-06, 9.77E-06)	3344 (2947, 3741)

The second set of simulations compared the performance between our ABC-MCMC implementation and the Bayesian MCMC approach implemented in the software Bayesian Evolutionary Analysis Sampling Trees (BEAST) [8].

Based on the results of the first simulation analysis, one hundred datasets were simulated using Bayesian Serial SimCoal [29]. We choose the following parameters for this simulation, there were three timepoints with inter-timepoint intervals set at 400 generations, the number of sequences per timepoint was fixed at 40, the mutation rate was fixed at 10^−5^ mutations per site per generation, and the effective population size was fixed at 3000.

For our LS and ABC-MCMC methods, only the relative frequency of nucleotides per site was available as data. For BEAST analyses, the simulated full-length sequence alignment was used. BEAST ran for 10 million iterations, and ABC-MCMC ran for 1 million iterations with 100,000 thousand iterations in the pre-processing stage. For both BEAST and ABC-MCMC, three independent chains were run for each dataset to check for convergence. Samples were recorded every 1000 iterations in order to reduce the autocorrelation and the first 10 % of the samples were discarded as burn-in. The trace plots were checked manually for convergence and the 95 % Highest Posterior Density region (HPD) for each parameter was calculated using Tracer [30].

## Results

### Simulation result 1: least square estimation

A series of simulations with different parameter settings were tested. Table 1 reports the means of LS estimates of population size and mutation rate obtained over 100 simulations. The results demonstrate that LS estimation requires sufficient numbers of sequences to obtain reasonable estimates of the proportion of each nucleotide for each site. When the number of full-length sequences is low (*n* < 10), change in one nucleotide at a site has a major effect on the nucleotide frequencies at that site; therefore, it is difficult for the LS algorithm is to estimate the true parameters. In contrast, as the number of sequences in the sample increases, estimation improves markedly.

In Tables 2 and 3, the number of generations between two consecutive timepoints has only a minimal effect on the estimation efficiency, hence all simulations with inter-timepoint intervals longer than 200 generations have similar performances. The number of timepoints does not have a major effect either.

**Fig. 2:**
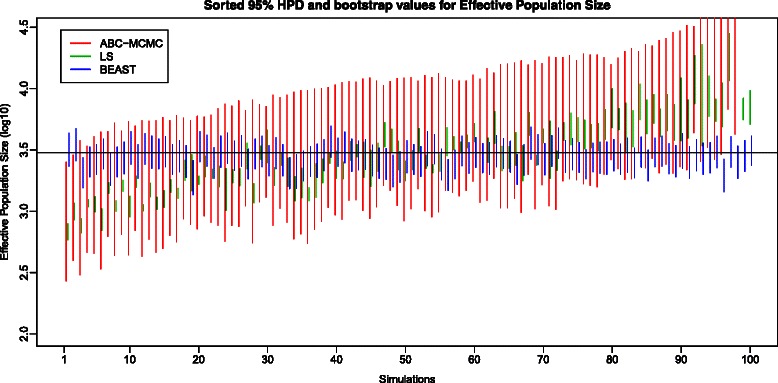
Plot of 95 % Confidence Intervals and 95 % Highest Posterior Densities of population size recovered using the LS bootstrap, ABC-MCMC and BEAST. The green lines are the 95 % CIs of LS bootstraps, the red lines are the 95 % HPDs of ABC-MCMC, and the blue lines are the 95 % HPDs obtained using BEAST. The true value of population size is shown as a solid black line. Note that the vertical axis is measured on a log scale

**Fig. 3:**
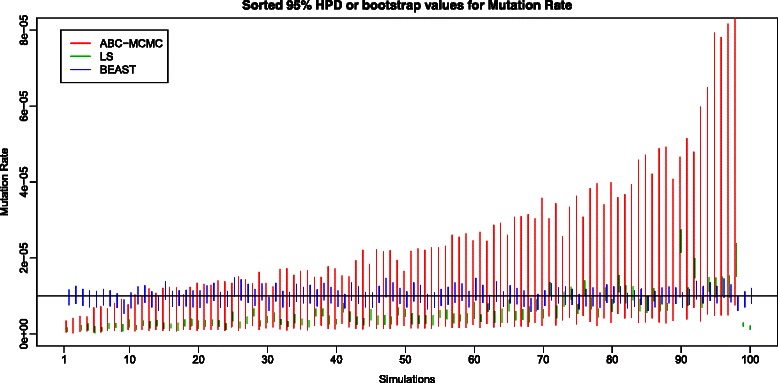
Plot of 95 % Confidence Intervals and 95 % Highest Posterior Densities of mutation rate recovered using the LS bootstrap, ABC-MCMC and BEAST. The green lines are the 95 % CIs of LS bootstraps, the red lines are the 95 % HPDs of ABC-MCMC, and the blue lines are the 95 % HPDs obtained using BEAST. The true value of mutation rate is shown as a solid black line

The relative efficiency of LS against the other two methods (ABC-MCMC and full-length Bayesian MCMC with BEAST) can be compared with the second set of simulations, where data were generated with 3 timepoints, with 40 sequences per timepoint and with intra-timepoint intervals of 400 generations. In these simulations, comparison of bootstrap 95 % Confidence Intervals of population size (Fig. 2) and mutation rate (Fig. 3) estimates, however, revealed an unflattering picture of LS estimation performance. Only a few LS 95 % Confidence Intervals enclose the true parameter values. Although the LS estimates are unbiased (i.e., their average over all simulations equals the true value), any given estimate performs poorly.

### Simulation results 2: BEAST and ABC-MCMC estimation

BEAST was used to analyze 100 datasets with a constant-sized coalescent model with the parameters described above, to obtain estimates of both population size and mutation rate. Each analysis ran for 10 million iterations initially, and samples were stored every 1000 iterations. After manually inspecting the trace plots for convergence, some dataset were re-analysed with 100 million iterations. The 95 % HPD for mutation rate contains the true value 93 times and effective population size 89 times.

ABC-MCMC analyses were performed on the same 100 datasets with 1 million iterations and samples were stored every 1000 iterations. In addition, another 100,000 iterations of preprocessing simulations were used to estimate the sufficient statistics. An example of the regression equations used as sufficient statistics is in.

**Fig. 4:**
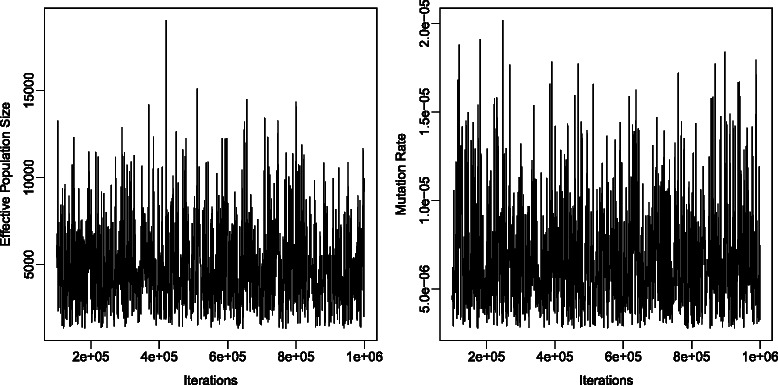
Trace plot from ABC-MCMC for both effective population size and mutation rate after removing the first 10 % of the generations as burn-in. This demonstrates that the MCMC chain mixes well

**Fig. 5:**
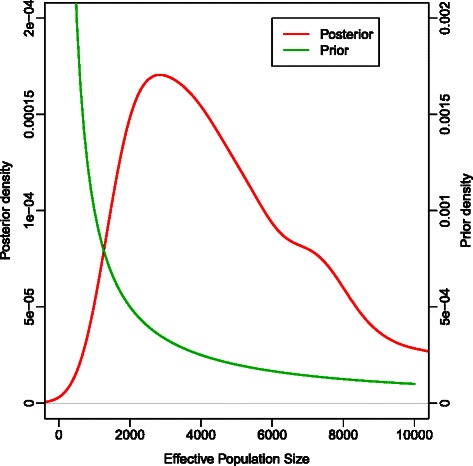
The prior and posterior distributions for the effective population size from ABC-MCMC

Some datasets failed to converge for ABC-MCMC and were reanalyzed with 10 million iterations. Two out of 100 datasets failed to converge even with 10 million iterations, and these are excluded from the analyses. Figure 4 is an example of the trace plot for both mutation rate and effective population size. The plot indicates that the MCMC chain mixes well. Figure 5 gives an example of the posterior density of the effective population size recovered, plotted against the prior distribution. Given the difference between the prior and posterior density, it is apparent that there is sufficient signal in the data to shift the posterior distribution of effective population size away from the prior distribution.

The 95 % HPD for mutation rate included the true value 87 times out of 98 analyses, and the 95 % HPD for population size included the true value 91 times out of 98 analyses. The results of both BEAST and ABC-MCMC are summarized in Table 4. Based on the number of 95 % HPDs that include the true values, both BEAST and ABC-MCMC perform similarly. However, the actual 95 % HPD intervals from ABC-MCMC are wider than the 95 % HPD from BEAST (see Figs. 2 and 3): on average the 95 % HPDs for effectively population size are 8-fold wider and those for mutation rate are 5-fold wider.

**Table 4:** Summarized the number of times that 95 % HPD includes the true value for both ABC-MCMC and BEAST

Algorithm	ABC-MCMC	BEAST
No. of Converged Dataset	98	100
No. of 95 % HPD for μ includes the true value (1e-5)	87	93
No. of 95 % HPD for N includes the true value (3000)	91	89
Mean Mutation Rate (Lower quartile, upper quartile)	8.367e-06 (5.892e-06, 1.443e-05)	9.730e-06 (9.104e-06, 1.040e-05)
Mean Effective Population Size (Lower quartile, upper quartile)	2457.4 (1503.0, 3637.9)	2851.688 (2623.965, 3134.936)

## Discussion and conclusions

In this paper, we propose two new algorithms to estimate evolutionary genetic parameters by using only the frequency of nucleotides at each site as input. The least-squares method provides a fast way to estimate effective population size and mutation rate, but our results indicate that LS estimates (and their bootstrap confidence intervals) tend to under- or over-estimate the true parameters. Consequently, we cannot recommend our LS algorithm as a standalone method for obtaining estimates of evolutionary parameters. Our LS method, nonetheless, provides a useful baseline for the training region which we need to use to derive nearly sufficient statistics for our ABC-MCMC procedure.

Parameter values recovered by ABC-MCMC are comparable to those obtained using BEAST, albeit with much wider 95 % HPDs. The performance of ABC-MCMC, relative to that of BEAST is unsurprising, because BEAST has access to the full-length alignment of all sequences in the sample. When the full-length alignment is summarized by the proportion of nucleotides at each site, there is inevitably a loss of information. Additionally, our methods do not reconstruct phylogenies of the sequences, further reducing estimation efficiency. Despite these significant constraints, it is interesting that the sitewise nucleotide frequencies are able to provide enough information to obtain meaningful estimates of the parameters of interest.

The methods we have developed assume that the reference sequence is available and short reads can be aligned to the reference accurately. Obviously the performance of this approach is dependent on the quality of the reference sequence and how well these short reads are aligned to it. As noted above, in our simulations we have obtained site nucleotide frequencies from the full-length sequences; we expect that with real NGS data, the accuracy with which we estimate the sitewise frequencies of each nucleotide will depend on sequencing error. Preprocessing the raw reads for quality, read-length, and identity to the reference sequence is likely to remove a significant amount of this error. In this case, it is unlikely that any remaining errors will distort the site frequencies enough to have a noticeable effect on the estimates. Also, all insertions and deletions (indels) have been ignored in all simulations and summary statistics. However, our implementation of the ABC-MCMC model allows indels at each site as a fifth “nucleotide” or state. We have not yet applied this to our analysis.

One major advantage of using only the site frequencies is that it can be applied to an arbitrary number of sequences in the original sample. In our simulations, we did not simulate short-read sequences; instead, we used the full-length sequences to derive the nucleotide frequencies at each site. Nonetheless, the amount of time required to estimate the proportion of each nucleotide for each site will scale linearly to the number of short-read sequences only, regardless of the number of full-length sequences from which these were derived, and it is likely to be a very fast calculation. In contrast, methods that rely on building phylogenies from full-length alignments must contend with the superexponential growth in the number of possible trees as the number of sequences increases. For a realistic NGS dataset, the number of reconstructed full-length haplotypes can be large. Consequently, our methods can be used on NGS datasets with large numbers of short read fragments, obtained from a large number of full-length sequences.

In this paper, we have only developed methods to estimate effective population size and mutation rate. Population geneticists are also interested in other evolutionary parameters, including migration rates and recombination rates. We believe that ABC-MCMC, like other Bayesian MCMC methods, provides a flexible framework to construct more complex evolutionary models. But the use of sitewise nucleotide frequencies alone means that we lack the finer-grained information afforded by a genealogy or phylogeny of full-length sequences; consequently, we are not certain how much complexity can be added to the models before the sitewise nucleotide frequencies we use in our methods lose all signal. This is certainly an area that we intend to explore. The source code for ABC-MCMC is available at https://github.com/stevenhwu/SF-ABC.
